# Remembering the impact of our collective voice

**DOI:** 10.1017/cts.2017.18

**Published:** 2017-08-10

**Authors:** Emma A. Meagher

**Affiliations:** 1 President, ACTS; 2 Vice Dean and Chief Clinical Research Officer, Perelman School of Medicine, University of Pennsylvania, Philadelphia, PA, USA

On May 2, 2017, National Institutes of Health (NIH) Director Francis Collins, M.D., Ph.D., announced a “new approach to making sure that we are exercising optimum stewardship of the funds we receive from tax payers.” The core element of the Grant Support Index (GSI) metric proposal was to place a cap on the number of grants an investigator may hold with the intent being to free-up funding for early-career investigators. Upon initial announcement, there was tentative support from some quarters. However, as the details underwent closer scrutiny, concerns began to be voiced. The motivation for the policy was clear to most and was felt to be warranted. Specifically, for ACTS, a part of whose mission is to cultivate the next generation of clinical and translational scientists, the motivation was closely aligned. The approach, however, was not.

The collective wisdom and voice of the engaged public, elected representatives, the NIH, and those it funds has affirmed, and, hopefully, will continue to do so, that biomedical research is essential. A congressional bipartisan collegiality is key in this regard and, thankfully, currently exists.

In that vein and in response to the NIH announcement, academic institutions, leaders of professional academic associations and societies such as ACTS, AAMC, CR Forum, among others, and investigators alike, rapidly began to evaluate the potential broad-spectrum impact of the policy and to voice their opinions. As the credible rhetoric of the scientific community gained momentum and the opinions regarding the potentially negative impact of the GSI policy were heard, carve-out exceptions to the cap (training grants, for example) began to evolve. Ultimately, on June 5, it was communicated that although the NIH would not be moving forward with plans to implement the GSI metric policy, it was committed to the implementation of alternative measures that would strengthen NIH funding support for early- and mid-career investigators.

This is an important example of the partnership between the community of biomedical scientists and the institution that funds the enterprise. It is also an exemplar of the importance of the impact of our own individual advocacy. A similar recent example was the overwhelming stakeholder response to the Advanced Notice of Proposed Rulemaking to the Common Rule that was equally impactful. We can be successful in impacting policy when the legitimacy of our viewpoints is heard.

Now we face a potentially new challenge from the fact that NIH/NCATs is deciding to reduce the current number of funded Clinical and Translational Science Awards (CTSAs) from 64 to 57, reduce the number of years of funding provided to some institutions, and/or impose percentage cuts to committed funds despite the recent increase in the NCAT FY2017 budget for the CTSA program. CTSA hubs are core programs in academic institutions that support the training of the next generation of clinical and translational scientists and provide the infrastructure that supports their work. Each hub represents far more institutional resources than NIH supplies, but the grant itself is the crucial catalyst for this important transformation of biomedical research in this country. Although the focused advocacy missions of CR Forum and ACTS continue, it is time that we again remember the power of our individual voices, and work with our Congressional, Administrative, and institutional partners to collectively ensure the vitality of the enterprise and, in doing so, strengthen the biomedical workforce and the enterprise.

